# Evaluation of the Role of Trace Elements in Malignant Transformation of Oral Submucous Fibrosis: A Study in the Population of Krishna District, Andhra Pradesh, India

**DOI:** 10.7759/cureus.65314

**Published:** 2024-07-24

**Authors:** Mary Sujatha Mekala, Ramesh Kumar Koothati, Komali Mummidi, Anuradha Chennupati, Reshma Priyanka Danam, Divya Harika Pedada, Kammacheruvu Jayaraja Amulya, Himaja Reddy Modeem

**Affiliations:** 1 Oral Medicine and Radiology, Government Dental College and Hospital, Vijayawada, IND; 2 Dentistry, Government Dental College and Hospital, Vijayawada, IND; 3 Oral Medicine and Radiology, Panineeya Institute of Dental Sciences and Research Centre, Hyderabad, IND

**Keywords:** malignant transformation, oral cancers, oral malignancy, oral sub mucous fibrosis, trace elements

## Abstract

Introduction: Oral submucous fibrosis (OSMF) is a chronic, potentially malignant disorder linked predominantly to areca nut chewing. This study investigates the role of serum trace elements (Cu, Fe, Zn, Se) in the progression and malignant transformation of OSMF.

Methodology: A community-based cross-sectional study involved 80 participants from the Government Dental College, Vijayawada, Krishna District, AP. Participants were divided into four groups: areca nut users without OSMF, areca nut users with OSMF, areca nut users with malignant-transformed OSMF, and healthy controls. Blood samples were analyzed for serum iron, copper, zinc, and selenium levels.

Results: Significant differences were found in serum copper (P=0.032) and zinc levels (P=0.006), with elevated levels observed in the malignant-transformed OSMF group compared to controls. No significant differences were observed in serum iron (P=0.542) and selenium levels (P=0.062) across the groups.

Conclusion: Elevated serum copper levels can serve as a reliable diagnostic marker for malignant transformation in OSMF. Future research should explore the potential therapeutic benefits of copper chelation in OSMF management.

## Introduction

Oral submucous fibrosis (OSMF) is a chronic and potentially malignant disorder affecting any part of the oral cavity [1]. This condition is primarily linked to the habitual chewing of areca nut (often referred to as betel nut), which is a common practice in many Asian countries. OSMF presents a range of symptoms, including blanching of the mucosa, restricted mouth opening, burning sensation, and increased mechanical stiffness of mucosa [2,3]. The global prevalence of oral malignancy, which ranks as the sixth most common cancer worldwide, points out the seriousness of OSMF due to its potential to transform into oral cancer. The high mortality and morbidity rates associated with oral cancer highlight the critical need for early detection and intervention [4]. Early detection of potentially malignant disorders like OSMF can significantly reduce mortality and morbidity. One promising approach for early detection is monitoring alterations in the concentrations of trace elements in body fluids, particularly blood serum. Trace elements such as copper (Cu), iron (Fe), zinc (Zn), and selenium (Se) often show altered levels in individuals with precancerous conditions. These biochemical changes in the serum can serve as important biomarkers, aiding not only in the early diagnosis and treatment of OSMF but also in assessing the prognosis of the condition [5].

Objective

Primary Objective

A comprehensive evaluation of the role of serum trace elements in various functional and clinical gradings of OSMF, along with OSMF associated with carcinoma, and the estimation and comparison of the serum levels of trace elements in OSMF patients with habit association, individuals with a history of habit association but not suffering from OSMF, those where OSMF has transformed into malignancy, and healthy individuals with no habits.

Secondary Objective

Assessment of a reliable diagnostic biomarker among the trace elements in determining malignant transformation of OSMF.

## Materials and methods

A community-based cross-sectional study was conducted on the individuals attending Out Patient Department of Oral Medicine and Radiology with a history of areca nut/betel quid/*gutkha* chewing for more than one year at Government Dental College, Vijayawada, Krishna District, AP. The study was approved by the Institutional Ethics Committee (Ref. No.7/IEC-GDCH/2022 ), Government Dental College and Hospital, Vijayawada, Krishna District, AP. The study followed the ethical principles of the Declaration of Helsinki and the World Medical Association’s code of ethics. The nature of the study is explained, the informed consent of all the patients included in the study is obtained in written form, and their confidentiality is preserved.

A total of 80 participants were divided into four groups: Group I consisted of 20 individuals with a history of habit association without any evidence of OSMF; Group II included 20 individuals with a history of habit association with evidence of OSMF; Group III comprised 20 individuals with a history of habit association with malignant transformation of OSMF; Group IV had 20 apparently healthy individuals without any habit association were taken as controls.

The inclusion criteria were a positive history of areca nut, *gutkha*, or betel quid chewing, clinical evidence of OSMF (presence of fibrous bands, burning sensation, restricted mouth opening), and histologically proven OSMF with malignancy. Exclusion criteria included unwillingness to participate, a history of systemic disease or treatment, prior OSMF treatment, and individuals with the presence of other potentially malignant disorders.

A thorough history will be taken from each patient in their local language, and clinical examinations of the patients will be performed. OSMF/OSMF associated with malignancy is confirmed on the basis of clinical and histopathological reports and is graded based on Khanna and Andrade Classification [6]. A 5 ml venous blood sample was obtained and centrifuged, and the serum was collected in sterile vacutainers for trace element analysis: serum iron by the ferrozine method without deproteinization, serum copper by the 3,5-DIBR-PAESA method, serum zinc by the nitro-PAPS method, and serum selenium by ICP-MASS spectrometry.

Statistical analysis

Data regarding the age, sex, and values of serum trace elements falling into respective groups are collected, compiled, and analyzed using IBM SPSS version 2.0 software (IBM SPSS, IBM Corp., Armonk, NY, USA). Descriptive statistics, chi-square tests, and one-way analysis of variance with Tukey’s post hoc tests were done to analyze the study data. Bar charts were used for data presentation.

## Results

Age and gender distribution

The mean age of the entire study group was 41.3±10.92 years, and most participants in the study group were male (73.75%). One-way analysis of variance observed no significant difference (p= 0.234) in mean age between the study groups (Table 1).On comparison of gender distribution between the groups using the chi-square test, it was observed that the proportion of females in the no habit association with no OSMF group was significantly (p < 0.001) higher compared to the other three groups(Table 2).

**Table 1:** Age distribution in study groups OSMF: oral submucous fibrosis One-way analysis of variance; p≤0.05 considered statistically significant

Group	Number	Mean Age ± Standard Deviation	Standard. Error	F value	P value
Habit association + no OSMF	20	44.00 ± 11.603	2.595	1.453	0.234
Habit association + OSMF	20	38.75 ± 10.310	2.305		
Habit association + OSMF with malignant transformation	20	43.65 ± 7.393	1.653		
No habit association + no OSMF	20	38.80 ± 13.177	2.947		

**Table 2:** Gender distribution in the study group OSMF: oral submucous fibrosis Chi-square test; p≤0.05 considered statistically significant. *statistical significance

Group	Sex		P value
	Male N(%)	Female N(%)	
Habit association + no OSMF	17 (85)	3 (15)	<0.001*
Habit association + OSMF	18 (90)	2 (10)	
Habit association + OSMF with malignant transformation	17 (85)	3 (15)	
No habit association + no OSMF	7 (35)	13 (65)	

Evaluation of serum levels of trace elements in the study group

**Figure 1:**
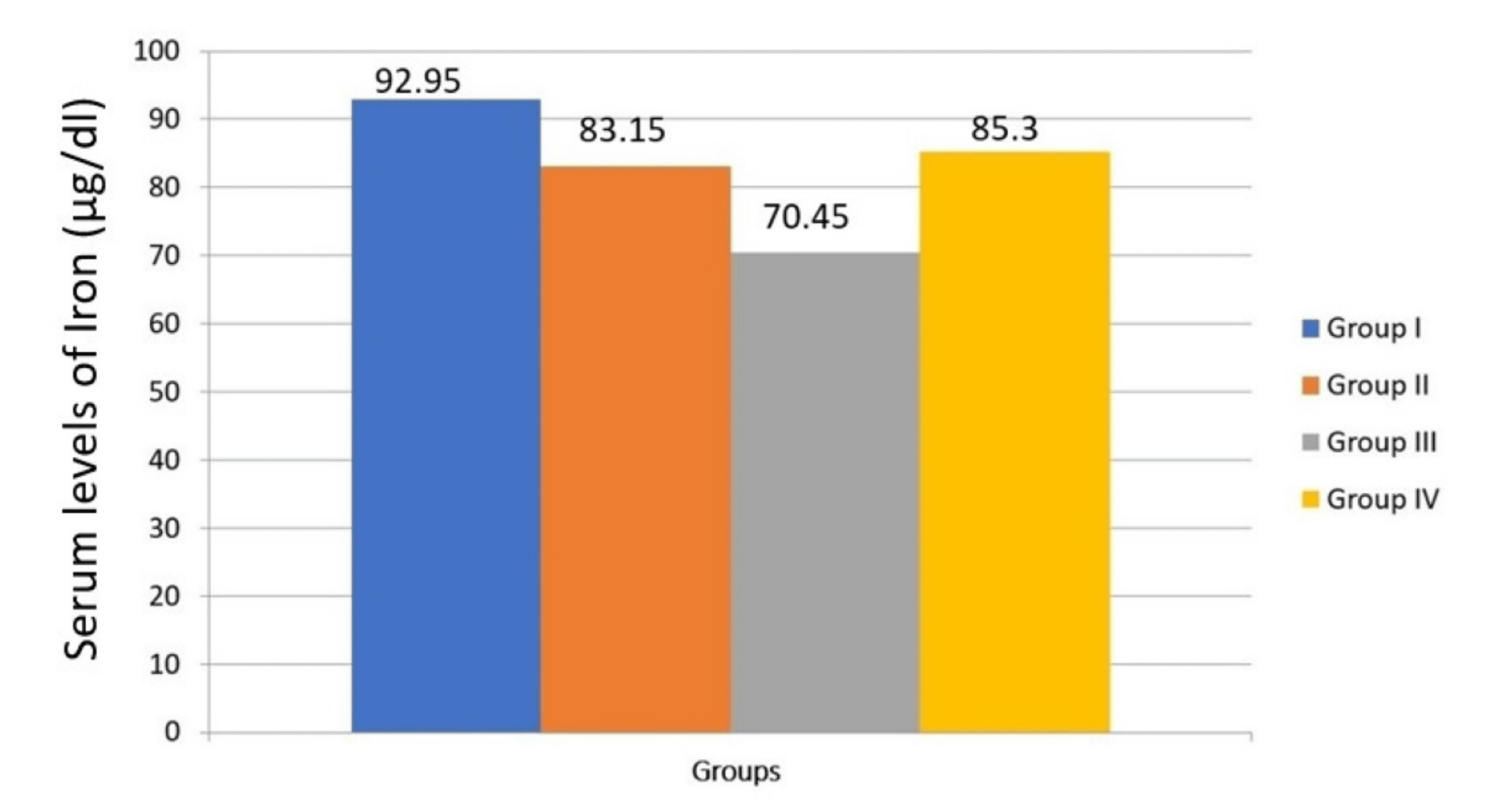
Comparison of serum levels of iron between the study groups

**Figure 2:**
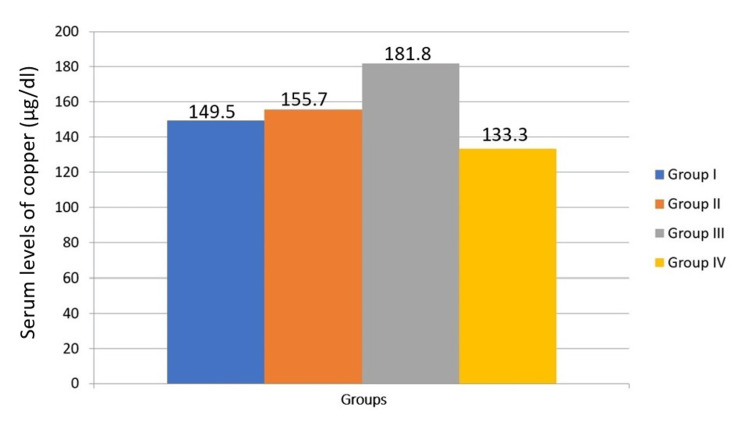
Comparison of serum levels of copper between the study groups

**Figure 3:**
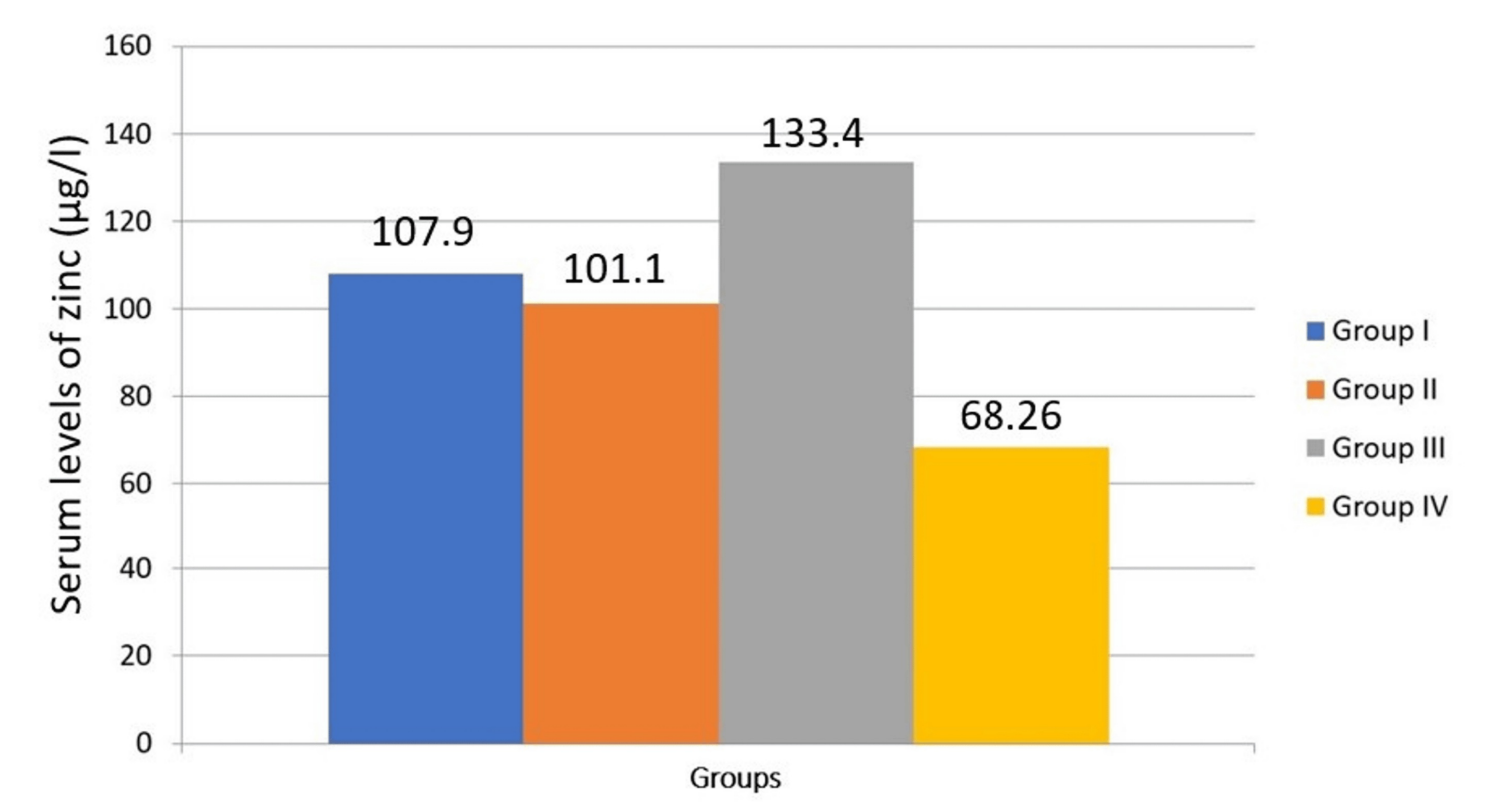
Comparison of serum levels of zinc between the study groups

**Figure 4:**
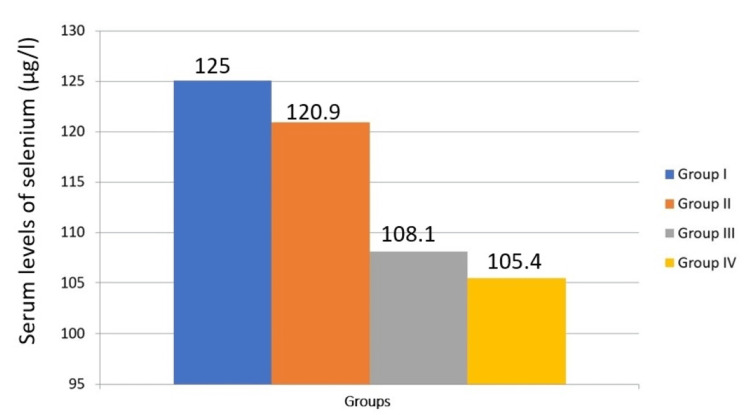
Comparison of serum levels of selenium between the study groups

The one-way analysis is used to compare serum levels of trace elements in the study group (Table 3). Although iron levels tend to decrease with disease progression, there is no significant difference in iron levels across the four groups (P = 0.542). This suggests that iron levels are relatively stable regardless of the presence of habits, OSMF, or malignant transformation (Figure 1). There is a significant difference in copper levels between the groups (P = 0.032). Copper levels tend to increase with the progression from habits to OSMF and further to malignant transformation (Figure 2). This indicates that copper could be a potential biomarker for disease progression. There is a significant difference in zinc levels between the four groups (P = 0.006). Zinc levels show variability but generally increase with malignant transformation (Figure 3). This suggests that zinc levels might be associated with the severity of the condition. There is no significant difference in selenium levels across the groups (P = 0.062). Although selenium levels tend to decrease with disease progression, the difference is not statistically significant (Figure 4). In post hoc analysis, for both copper and zinc levels, significant differences were identified between those with habit association and malignant transformation of OSMF and the control group.

**Table 3:** Comparison of serum levels of trace elements in the study groups OSMF: oral submucous fibrosis One-way analysis of variance; p≤0.05 considered statistically significant. *statistical significance; ^a^statistically significant differences in post hoc Tukey analysis

Parameter	Group	Number	Mean ± Standard Deviation	Standard Error	F value	P value
Iron	Habit association + no OSMF	20	92.9500 ± 41.25591	9.22510	0.721	0.542
	Habit association + OSMF	20	83.1500 ± 52.65706	11.77448		
	Habit association + OSMF with malignant transformation	20	70.4500 ± 50.94112	11.39078		
	No habit association + no OSMF	20	85.3000 ± 51.09856	11.42599		
Copper	Habit association + no OSMF	20	149.5385 ± 52.72437	11.78953	3.09	0.032*
	Habit association + OSMF	20	155.7625 ± 52.64418	11.77160		
	Habit association + OSMF with malignant transformation	20	181.823^a ^± 65.44421	14.63377		
	No habit association + No OSMF	20	133.365^a ^± 26.02897	5.82026		
Zinc	Habit association + no OSMF	20	107.9570 ± 76.44763	17.09421	4.49	0.006*
	Habit association + OSMF	20	101.1685 ± 55.79563	12.47628		
	Habit association + OSMF with malignant transformation	20	133.479^a ^± 61.21027	13.68703		
	No habit association + no OSMF	20	68.2695^a ^± 11.76819	2.63145		
Selenium	Habit association + no OSMF	20	125.0585 ± 17.32924	3.87494	2.361	0.062
	Habit association + OSMF	20	120.9010 ± 27.08268	6.05587		
	Habit association + OSMF with malignant transformation	20	108.1050 ± 32.05390	7.16747		
	No habit association + no OSMF	20	105.4790 ± 22.22877	4.97050		

## Discussion

The rapid spread of oral precancerous and cancerous lesions like an epidemic is alarming. The incidence of oral squamous cell carcinoma (OSCC) is notably higher among individuals with OSMF, making it one of the key precancerous conditions linked to OSCC.OSMF is a chronic, slowly progressing condition with potential for malignancy, often linked to areca nut consumption. Once limited to the Indian subcontinent, OSMF is now increasingly observed in Asian populations residing in the United Kingdom, USA, and other developed nations, posing a significant global health challenge. The major threat associated with OSMF is its potential to transform into malignancy, with reported transformation rates approximating 7.6% over a 17-year period [7].

OSMF manifests with symptoms such as dry mouth, burning sensation, pain, taste alterations, limited tongue mobility, trismus, and difficulty in swallowing. The oral mucosa becomes notably inelastic and opaque, displaying vertical bands that appear pale on inspection and firm on palpation [8]. In advanced stages, there is stiffening of oral mucosa and esophagus, resulting in reduced mouth opening and functional impairments such as difficulties in eating, maintaining oral hygiene, and speaking effectively [9]. Ultimately, OSMF can progress to oral cancer within 3 to 16 years following the initial diagnosis of OSMF [8].

OSMF is primarily linked to the consumption of areca nuts, with a clear correlation observed between the frequency and duration of areca nut chewing (without tobacco) and the development of OSMF. Commercially available freeze-dried products like pan masala, *gutkha*, and *mawa* (which contain high concentrations of areca nut per chew) appear to accelerate the onset of OSMF compared to traditional betel quid preparations, which typically contain smaller amounts of areca nut. Alkaloids present in areca nuts are believed to disrupt the molecular processes involved in the deposition and breakdown of extracellular matrix components such as collagen. In vitro studies conducted on human fibroblasts using areca nut extracts or purified arecoline provide evidence supporting the theory of increased fibroblast proliferation and collagen synthesis, which are also observed histologically in human OSMF tissues [10]. Additionally, there is evidence suggesting that areca nut reduces the phagocytosis of collagen by fibroblasts and may influence the expression of copper-dependent enzymes like lysyl oxidase, matrix metalloproteinases, and tissue inhibitors of matrix metalloproteinases. It is hypothesized that areca nut might induce OSMF by elevating levels of cytokines in the lamina propria, and genes related to collagen metabolism are implicated in the susceptibility and pathogenesis of OSMF [11].

In this study, the mean age of the study sample was 41.3±10.92 years, with the mean age of individuals with a history of habit association and OSMF group being 38.75, which is similar to studies conducted by Nigam et al. [12], who reported that most OSMF cases occurred in the 36-40 year age group and Srivastava et al. [13] who observed that OSMF is most common in individuals aged 30-40 years, followed by those aged 20-30 years. This may be attributed to the increased social interactions and economic freedom experienced during these ages in a rapidly developing country like India. Consequently, individuals in this age group are more likely to engage in chewing habits such as betel nut, *gutkha*, pan masala, smoking, and alcohol consumption, either as a fashion statement, to alleviate stress, or due to peer pressure.

In the current study, 59 participants (73.75%) were male. Comparing gender distribution across the groups revealed a significantly higher proportion of females in the control group compared to the other three groups (p<0.001). Additionally, there is a higher incidence of OSMF and its malignant transformation among men (85%-90%) compared to women (10%-15%). This finding is similar to studies by Yang et al. [14], Sinor et al. [15], and Srivastava et al. [13], who also reported a male predominance among OSMF cases. This male predominance might be attributed to easy accessibility for males to use areca nut and its products more than females.

In the current study, there is a gradual decrease in the mean value of serum iron in Group I (92.95), Group II (83.15), and Group III (70.45), indicating a correlation between the habit association, OSMF, malignant transformation of OSMF and serum iron in individuals which is similar to other studies [5,8,16] conducted across the world. Iron is crucial for the function of many enzymes, such as cytochrome oxidase, xanthine oxidases, succinate dehydrogenase, glucose-6-phosphate dehydrogenase, catalases, peroxidases, and choline dehydrogenase. It serves as a cofactor for prolyl hydroxylase (PH) and lysyl hydroxylase, enzymes essential for collagen hydroxylation. In OSMF, excessive collagen synthesis may lead to reduced serum and plasma iron levels. Additionally, the burning sensation and restricted mouth opening associated with OSMF limit food intake, exacerbating micronutrient deficiencies. Cytochrome oxidase, an iron-dependent enzyme, is necessary for epithelial development, and iron deficiency in OSMF patients reduces cytochrome oxidase levels, leading to epithelial atrophy [16]. Whereas, in the current study on the Krishna population, the serum iron mean value of the control group (85.3) is comparatively lesser than that of Group II individuals (92.95). This can be attributed to the significantly higher number of samples collected from the female population in the control group. According to the Fourth National Family Health Survey (NFHS-4), 54.4% of women of reproductive age are found to be anemic in India [17].

The current study in the Krishna population shows a significant increase in serum copper among individuals with a history of habit association and malignant transformation of OSMF than controls (P=0.032). Also, there is a gradual increase in the mean values of serum copper among individuals with an increased duration of history of habit association, which is similar to the results obtained by other studies [5,8,10,16,18] around the world. This is because copper plays a crucial role in red blood cell formation and collagen synthesis in bones and connective tissue and aids in iron absorption [18]. It is also vital for the function of enzymes such as Cu/Zn-superoxide dismutase and lysyl oxidase [19]. Trivedy et al. [20-22] have carried out a series of studies on the estimation of copper levels in areca nut, as well as in the serum and tissue of OSMF patients. They found that the copper content in areca nuts is higher than in nuts used in snacks commonly consumed. Copper regulates fibrosis production via the enzyme lysyl oxidase, which facilitates collagen crosslinking and makes it more resistant to enzymatic degradation.

According to the current study in the Krishna population, there is a significant increase in serum zinc levels in individuals with habit association and malignant transformation of OSMF than individuals in the control group (p=0.006), which is similar to results obtained in research by Khanna et al. [10], suggesting a consistent link between elevated serum zinc levels and the progression of OSMF. This emphasizes the importance of monitoring serum zinc levels as a potential biomarker for early detection and intervention in OSMF cases.

Safaralizadeh et al. [23] determined the normal serum selenium values in a healthy individual to be around 100.6 ± 13 SD μg/l. In this study on the Krishna population, the serum selenium mean values lie approximately in the normal range of all the individuals under Group I (125.0585), Group II (120.901), Group III (108.105), and Group IV (105.479), indicating that the role of selenium in OSMF and its malignant transformation is insignificant. Selenium, an antioxidant, is a crucial component of enzymes such as glutathione peroxidase, type I iodothyronine deiodinase, metalloprotein, fatty acid binding protein, and selenoprotein P [24]. While the role of oxidative stress is well-established in the pathogenesis of OSMF, estimations of individual entities that modulate the process may not be reflective of their proposed role. This may be due to the fact that the process of oxidative stress has many pathways, and the detection or expression of any one element or enzyme may not necessarily be involved.

Limitations of the study

The present study, while providing valuable insights into the role of serum trace elements in OSMF and its malignant transformation, has few limitations. The small sample size and focus on a single geographic location limit the generalizability of the study. Potential confounding variables such as diet, genetics, and lifestyle habits were not controlled, and significant gender disparity might affect the outcomes. The reliance on standard biochemical analysis methods could be improved with more advanced techniques like mass spectrometry, and the lack of longitudinal follow-up data restricts the understanding of trace element changes over time.

## Conclusions

In conclusion, this study demonstrates a significant association between serum trace element levels and the various stages of OSMF and its malignant transformation. Notably, there was an increase in serum copper and zinc levels in individuals with a history of habit association and malignant transformation of OSMF compared to healthy participants, suggesting these elements' potential role in disease progression. The elevated serum copper levels, in particular, highlight its reliability as a diagnostic marker for the malignant transformation of OSMF. Further research is warranted to explore the therapeutic implications of copper-chelating agents in preventing the malignant transformation of OSMF. The findings underscore the importance of monitoring trace element levels in individuals at risk, which could aid in early diagnosis and improved prognostic strategies for OSMF and its malignant transformation.
